# How to Attain Old Age

**Published:** 1854-01

**Authors:** 


In the latter years of his life he published an "*Earnest Exhortation*," which he closes by saying, "Since length of days abounds with so many blessings and favors, and I happen to be one of those who have arrived at that state, I cannot but give my testimony in favor of it; and I assure you all that I really enjoy more than I express, and that I have no other reason for writing, but that of demonstrating the great advantages which arise from longevity, to the end that their own conviction may induce them to observe those excellent rules of temperance and sobriety. And therefore I never cease to raise my voice, crying out to you, may your days be long, *that you may be the better servants to the Almighty*."

When about to die, he raised his eyes and exclaimed, with great animation, "Full with joy and hope, I resign myself to thee, most merciful God!" He then disposed himself with dignity, and closing his eyes, as if about to slumber, gave a gentle sigh and expired, in his ninety-ninth year, A.D. 1565.

If a systematic life of temperance has given sixty years additional to a broken-down constitution of forty, it becomes almost a crime for an invalid under fifty years of age not to avail himself of the trial.

This was a case where the energies of the stomach have been restored by temperance in eating and drinking, and remaining in their integrity for more than half a century thereafter; and what has been, may be again.

The next example shows that the stomach is made, *in modern times* too, to last a hundred and fifty years.

Thomas Parr, of Shropshire, England, when a hundred and twenty years old, married a widow for his second wife, who lived with him twelve years, and who stated that, during that time, he never betrayed any signs of age or infirmity. The King of England having heard of him, invited him to London in his hundred and fifty-second year. He was treated in so royal a manner at court, and his mode of living was so totally changed, that he died soon after, in 1635, aged one hundred and fifty-two years and nine months, proven by public documents. His body was examined by Dr. Harvey, who "found his internal organs in the most perfect state, nor was the least symptom of decay found in them. His cartilages, even, were not ossified, as is the case in all old people. The smallest cause of death had not settled in his body; and he died merely of phlethora, because he had been too well fed."

This man was a farm-servant, and had to maintain himself by daily labor, consequently he must have lived on plain food, and not over-abundant; and the simple fact that at his death his stomach was in a healthy condition, proves conclusively its capabilities of duration, working healthfully to the last. And there can be no reason, in the nature of things, why the human stomach may not be preserved in its integrity as a general rule to a like old age.

I trust no reader will attempt to live on common allowance, on his own responsibility; he should consult with his family physician, for age, sex, condition in life, occupation, materially modify the amount of food requisite for the wants of the system.

